# A journey to and through injury epidemiology

**DOI:** 10.1186/2197-1714-1-3

**Published:** 2014-03-20

**Authors:** Jess F Kraus

**Affiliations:** Department of Epidemiology, University of California, Los Angeles, California

**Keywords:** Injury epidemiology, Accident prevention, Traffic accidents, Occupational accidents, Safety

## Abstract

This brief commentary describes key events in the development of Dr. Jess Kraus’s professional career in injury epidemiology from the 1950s to the 2000s. It highlights the interactions with Dr. William Haddon Jr. and other researchers that were instrumental to his contributions to the field of injury epidemiology.

## Background

This Commentary briefly describes a few pivotal points in the development of my professional interest in injuries and their prevention. It began while employed with a rocket engine manufacturer in the late 1950s. I was invited to become a “safety engineer” not because of my college degree but because of some chemistry in my academic program. The major focus of the safety engineer was to keep the workers from killing themselves and unleashing a horrendous explosion. The major resource to the safety engineer at that time was in the industrial safety milieu which included the volume by Herbert Heinrich (1941). Unfortunately, research methods were not a part of this volume and, with only rudimentary skills in statistics, I sought the advice of Professor Alice Spillane who suggested a visit to the University of California, Berkeley. Dr. Charles Smith, Dean of the School of Public Health, thought that my interest in injuries was best served by enrollment in the Occupational Health Program. The introduction to epidemiology came from Professor Reuel Stallones who challenged me almost daily to look for the utility of epidemiology in injuries and their prevention. It soon became clear that this discipline could contribute much to the understanding and prevention of injuries. Thus, my career in Public Health was launched.

While at Berkeley I met Dr. Julian Waller who worked for the California State Department of Public Health. He was in my opinion one of the early giants in the field of injury research (e.g., Waller 1985, 1994). It was during a Health Department in-house conference on emergency transport that Dr. Waller stated, to a somewhat stunned audience, that it was safer to ride in a UPS delivery truck than in an ambulance. That got me thinking about what could be accomplished with some data on the frequency, characteristics, and outcomes following injury.

The literature on epidemiology and injuries before the 1960s was sparse with only a few American reports published by Drs. John Gordon and Helen Roberts both from the Harvard School of Public Health (Gordon 1949a,b, 1951; Roberts et al. 1952; Roberts and Gordon 1949). Their observations were expanded and improved by Dr. William Haddon Jr. who, with Dr. Edward Suchman and David Klein, published their ideas in the pioneering text “Accident Research: Methods and Approaches” (Haddon et al. 1964).

Following Berkeley, my family and I moved to Minneapolis at the invitation of Dr. Gaylord Anderson, Dean of the School of Public Health, University of Minnesota. There, in 1965, I first met Dr. William Haddon Jr. who was on a research grant site visit to the University’s Laboratory of Physiological Hygiene to evaluate a proposal to study eye movement during motor vehicle operation. Following the initial grant review session (in which he asked very direct and at times deprecating questions of the grant’s principal investigator), Dr. Haddon asked who I was as though I had no reason to be in the room. The first lessons from Dr. Haddon as a result of the site visit were “do not take anything for granted and make sure your conclusions are based on the data”. We talked briefly after the session and he asked about my plans after finishing the doctoral program. I recall telling him that with the brutal winters in Minneapolis and a wife and five kids, I looked forward to relocating to a warmer climate with a paying job, but if he did not mind I would like to contact him when I resettled. He said, "sure thing". Leaving Minneapolis was bitter sweet inasmuch as Professor Richard Bond, one of my advisors, and Professor Leonard Schuman, Chair of Epidemiology, invited me to stay on in a faculty position. I will not forget their guidance, for without it I might have followed a different path.

My first job after graduate school in 1968 was with the Public Health Service (PHS), Division of Accident Prevention in Cincinnati, Ohio. My unit (Accident Research) was staffed with a total of four people including me and one secretary. One accomplishment while with the PHS was the establishment of the groundwork for what today is the National Electronic Injury Surveillance System (NEISS) (Kraus 1972). Our small unit took pride to learn in later years that the pilot programs we introduced in three Public Service Hospitals would give rise to an effective way to gauge injury surveillance nationwide.

While with the PHS I learned more of Dr. Haddon’s theories and activities in his role as the first Director of the National Traffic Safety Agency and the National Highway Safety Agency [later combined as the initial Director of the National Highway Traffic Safety Administration (NHSTA)]. Later he was to become the first leader of the Insurance Institute for Highway Safety (IIHS).

Following the disbanding of the PHS Bureau of Community Environmental Management and the Division of Accident Prevention in 1969 (no doubt for political reasons), I was fortunate to land a job with the School of Medicine at the University of California, Davis. Although injury research was not the focus of my department I identified faculty in Orthopedic Surgery and Rehabilitative Medicine who wanted to work on aspects of injury. This was encouraging.

During the 1969 annual Meeting of the American Public Health Association (APHA), I met Dr. Haddon again, who, in his usual manner, asked what I was doing with my time. I explained that since there were virtually no sources for funding for injury work I would have to forgo research for awhile. Dr. Haddon asked what I had in mind. I explained that motorcycle crashes (yes, I used the word crashes not accidents) and deaths were constant headlines in the Sacramento newspaper and there was virtually no literature on the subject. He quizzed me on what I could offer to the problem and I explained that with access to mortality and crash data from the Sacramento County Coroner and the California Highway Patrol, respectively, epidemiologic analyses were possible but, I would need some funding. He asked that I send him a brief proposal and budget. I did by letter and received, about a week later, a check for the requested amount and a note wishing me good luck. This “grant” was not without difficulty since I had failed to submit the funding request through university channels including provisions for indirect costs. But a good hearted Associate Dean allowed the “gift” to go through but warned me about future endeavors from private sources.

This award from the IIHS was the first of many over decades. Dr. Haddon (and later Brian O’Neill) was always open to new research topics (e.g. refrigerator entrapment and brain injury) and, to my relief, seldom if ever argued over the amount of funding. Dr. Haddon’s application of epidemiological research methods to questions of the day served as a great model for me. His early case–control studies on pedestrian fatalities (Haddon et al. 1961), auto crashes (McCarroll and Haddon 1962) in New York as well as his study of nonfatal injuries among snow skiers (Haddon et al. 1962) are without doubt, benchmark accomplishments.

In reflection, without Dr. Haddon’s support and that of Brian O’Neill, and the collaboration of Drs. Leon Robertson and Alan Williams, I may not have followed an injury research path nor would I have had the opportunity to develop a dedicated research center at University of California, Los Angeles and venture into the area of neurotrauma research.

As a young faculty member at University of California, Davis, I was encouraged to join professional societies or associations connected with my research interests. The American Public Health Association (APHA) was a natural choice and its Epidemiology Section was a venue for presentation and discussion of injury research. Unfortunately, there were very few APHA members who shared interest in injury as a Public Health problem. In 1978, I was invited to chair the Program Committee of the Epidemiology Section. My thought for the next year’s conference was to highlight accomplishments of Epidemiology in Public Health in the first 50 years of the Section’s history (1929–1979). The Program Committee came up with the notion of surveying the section members asking who or what in their view had been the 10 most important epidemiological accomplishments in those first 50 years.

The survey results were quite surprising not because of the recognition of Drs. Carlton Gagdusek and Baruck Blumberg for their 1976 Nobel Prize work on slow viruses and Hepatitis B, respectively, but Dr. Haddon was voted among the top 10 contributors. When I phoned Dr. Haddon with the news and an invitation to be a keynote presenter at the 1979 annual meeting he said that he did not believe the results and never thought Public Health would ever embrace injury. I sent him the actual ballots and he agreed to give the presentation which can be found in the 1980 issue of Public Health Reports along with those of the remaining contributors (Haddon 1980).

It was about that time that Dr. Haddon introduced me to Sue Baker (another giant in the field). Her work and publications had been described frequently by Dr. Haddon. Unfortunately, the coast to coast distance was a major impediment to any collaboration despite our best intentions.

In the summer of 1980 I transferred to the Department of Epidemiology in the School of Public Health at the University of California, Los Angeles. School administration and faculty of the department were very supportive of my nontraditional research namely, injuries. In the mid-1980s a giant step in the application of epidemiology in injury research occurred with a focus under the umbrella of the Centers for Disease Control (CDC). Funding was available for epidemiological studies and later as part of academic centers supported by CDC. I felt very fortunate to have secured CDC funding for the Southern California Injury Research Center and as a result was able to meet and interact with many accomplished national and international researchers. I had also the great pleasure of mentoring many master’s and doctoral students while at the University of California, Los Angeles, many of whom have gone on to carry the torch of injury epidemiology.

## Conclusions

In sum, the global interest in injury research and epidemiology did not emerge overnight. The movement, if you will, began with an eclectic group of engineers, social and behavioral scientists, physiologists, physicians, educators and many others all who were aghast with the carnage resulting from crashes on our streets and highways. The call for a Public Health effort in prevention began with the work of Drs. John Gordon and Helen Roberts in the late 1940’s and continues today. I am proud to have played a small role in its development.
